# Energy and exergy analysis of a novel turbo-compounding system for supercharging and mild hybridization of a gasoline engine

**DOI:** 10.1007/s10973-020-10178-z

**Published:** 2020-09-03

**Authors:** Farhad Salek, Meisam Babaie, Ali Ghodsi, Seyed Vahid Hosseini, Ali Zare

**Affiliations:** 1 Faculty of Mechanical and Mechatronic Engineering, Shahrood University of Technology, Shahrood, Iran; 2 School of Science, Engineering and Environment, University of Salford, Manchester, UK; 3 Flow, Aerosols and Thermal Energy (FATE) Group, School of Engineering, Deakin University, Melbourne, VIC 3216 Australia

**Keywords:** Air Brayton cycle, Waste heat recovery, Hybridization, Supercharger, Turbo-compounding, Exergy, Downsizing

## Abstract

Number of hybrid vehicles has increased around the world significantly. Automotive industry is utilizing the hybridization of the powertrain system to achieve better fuel economic and emissions reduction. One of the options recently considered in research for hybridization and downsizing of vehicles is to employ waste heat recovery systems. In this paper, the addition of a turbo-compound system with an air Brayton cycle (ABC) to a naturally aspirated engine was studied in AVL BOOST software. In addition, a supercharger was modeled to charge extra air into the engine and ABC. The engine was first validated against the experimental data prior to turbo-compounding. The energy and exergy analysis was performed to understand the effects of the proposed design at engine rated speed. Results showed that between 16 and 18% increase in engine mechanical power can be achieved by adding turbo-compressor. Furthermore, the recommended ABC system can recover up to 1.1 kW extra electrical power from the engine exhaust energy. The energy and exergy efficiencies were both improved slightly by turbo-compounding and BSFC reduced by nearly 1% with the proposed system. Furthermore, installing the proposed system resulted in increase in backpressure up to approximately 23.8 kPa.

## Introduction

Air pollution is one of the major challenges that many countries are envisaged today. Transportation sector among the sources of emissions is responsible for about 40% CO_2_ emission around the world [1]. Apart from CO_2_, other harmful emissions such as particulate matters, CO, HC and NOx are emitted from the vehicle tailpipes running by fossil fuels. Even in recent COVID-19 pandemic, a direct relationship between the number of death and air pollution was observed [2]. Governments around the word have started to respond to this threat through international commitment such as Paris agreement and emissions regulations [3], and Europe has set probably the most stringent standards targeting reduction of about 4% CO_2_ yearly until 2030 [4].

Internal combustion (IC) engines burning fossil fuels were running the industries and transportations for many years. While it would be impossible to wipe off the IC engines completely in the near future, continuous improvements in fuel economy are required in automotive industry to achieve the vital targets set by standards [5–9]. To achieve this goal, researchers have been working on different solutions around the world such as using biofuel [10–15] and hydrogen energy [16, 17] as well as the hybridizations and electrifications [18–20] to reduce the engine fuel consumption and emissions. However, still further works are required to reduce the emissions from vehicles.

Approximately between 40 and 60% of fuel energy is wasted in IC engines through the exhaust [21, 22]. It is a huge share of energy especially considering the low efficiency of IC engines, and there is a great potential for recovering at least some of this wasted energy. Therefore, adding waste heat recovery (WHR) systems to IC engines could be viable in achieving a better fuel economy as it can result in increment of total engine power production and reduction of engine brake specific fuel consumption (BSFC) [21, 23, 24]. Moreover, extra electrical power will be generated by WHR systems which can be consumed directly or auxiliary in the vehicles.

There are many types of thermodynamic cycles which can be coupled to engines as the waste heat recovery system [25–29], and the most well-known one is the organic Rankine cycle (ORC) [21, 27]. This cycle contains four components in which the organic working fluid circulates in a cycle [22]. One of the most important thermophysical properties of organic fluids are their low heat of evaporation compared to other fluids, and they can be vaporized by absorbing heat from low-temperature heat sources [22]. Mahmoudi et al. [21] have reported that between 21 and 25% of engine exhaust waste heat can be recovered by the employment of recuperative single-loop and dual-loop ORCs, respectively. Salek et al. [22] have also studied the impact of ORC integrating for an internal combustion engine and showed that ORC was capable of recovering nearly 10% of wasted heat from the engine exhaust in a turbocharged diesel engine. Additionally, the CFD analysis of turbomachines used in ORC-WHR system was performed by different researchers to find the efficient solutions for increasing the thermal efficiency of the WHR [30].

Air Brayton cycle (ABC) is another cycle also recommended for WHR in the literature [24, 28, 29] which may have some advantages over ORC for vehicle engine applications as it is less complex. The lower number of ABC components when compared to ORC means that ABC will add less mass to vehicle than ABC in implementations. [31–33]. In addition, ORC needs its own working fluid, while the engine air can be used in ABC as the working fluid. Therefore, it seems using ABC in passenger cars is more beneficial than ORC.

The impacts of air Brayton cycle coupling to engine have been studied in the literature [34–37]. Nadera et al. [34] have investigated the fuel consumption reduction by employing ABC as the waste heat recovery system in a turbocharged gasoline fueled engine. In this study, part of the engine exhaust energy was absorbed by ABC heat exchanger for heating the ABC working fluid before entering the turbine. Based on the results of their work, vehicle fuel consumption decreased between 5.5 and 7% by using such waste heat recovery system. Song et al. [35] studied the performance of ABC coupled with a turbocharged diesel engine running at various engine speeds. An extra short fraction of the engine inlet air was also shared with Brayton cycle, and it is heated by the heat exchanger installed in engine exhaust system. The results showed that the engine fuel consumption decreased between 2.6 and 4.6% at different engine speeds. In another similar study [37], coupling ABC to a turbocharged diesel engine led to ~ 0.64 kW power recovery at engine rated RPM.

Analysis of thermodynamic systems using exergy will provide a comprehensive insight into the system performance and losses. While energy “cannot be created or destroyed” (first law), exergy can be lost or destroyed during different process due to the irreversibilities (second law). Employing second law of thermodynamics (exergy analysis) will reveal the energy degradations process within the system that cannot be understood from the first law (energy) analysis [38]. The exergy analysis was employed in different energy systems including the engine research to highlight the engine improvements and losses by alternation in engine and/or fuel [16, 39–43]. When exergy analysis was employed in literature, it was mainly for commonly used ORC cycle for waste heat recovery of heavy-duty engines [40, 41, 43]. Valencia eta al. [40] investigated the impacts of coupling ORC on BSFC and exergy efficiency for a 2 MW natural gas engine. Based on their results, BSFC decreased by nearly 7.67% at engine rated RPM. Salek et al. [43] have also studied a similar system in which a Kalina cycle was employed as the waste heat recovery system. It is reported that system exergy efficiency can be increased between 7.2 and 7.9% by integration of the waste heat recovery system. On the other hand, turbo-compounding systems have been widely used for small high RPM engines which are used in vehicle propulsion systems because of their low mass [44, 45]. A novel turbo-compounding system was introduced by Zi et al. [42] consisting of electric-booster and turbo-generator system. The engine waste heat was converted to electrical power by a turbine installed on engine exhaust system. The generated electrical power by turbine was used by electric-booster for supercharging the engine. As reported, the produced power by turbine can be managed in their system and exergy efficiency of vehicle propulsion system was increased by nearly 0.8%.

As demonstrated, the focus of literature on using WHR system was on ORC and turbocharged engines with the high exhaust gas pressure. However, the benefits of installing the ABC on naturally aspirated gasoline fueled engine have not been studied. Furthermore, the exergy analysis can help in better understanding of the effectiveness of the WHR systems. Therefore, in this paper, the ABC was studied as the WHR system for hybridization and turbo-compounding of a naturally aspirated sport gasoline fueled engine. The exergy analysis was employed to explain the effect of adding turbo-compounding system on the exergy performance parameters of the whole system. To develop this study, the experimental data were collected from the original engine to benchmark its performance. Then, the engine was modeled in AVL BOOST software, and the model was validated against the experimental data. Finally, the ABC was integrated into the engine to demonstrate the energy recovery from the waste heat through the exhaust. The recovered power was used to boost the air into the engine for producing more power, and the performance of engine has been evaluated at rated engine speed. A throttle was recommended for proposed ABC design to control the fraction of air entering the ABC heat exchanger. The exergy analysis has been performed to understand the effectiveness of adding ABC to the engine and finding the exergy destruction of the components.

## Methodology

### Experimental set-up

**Fig. 1:**
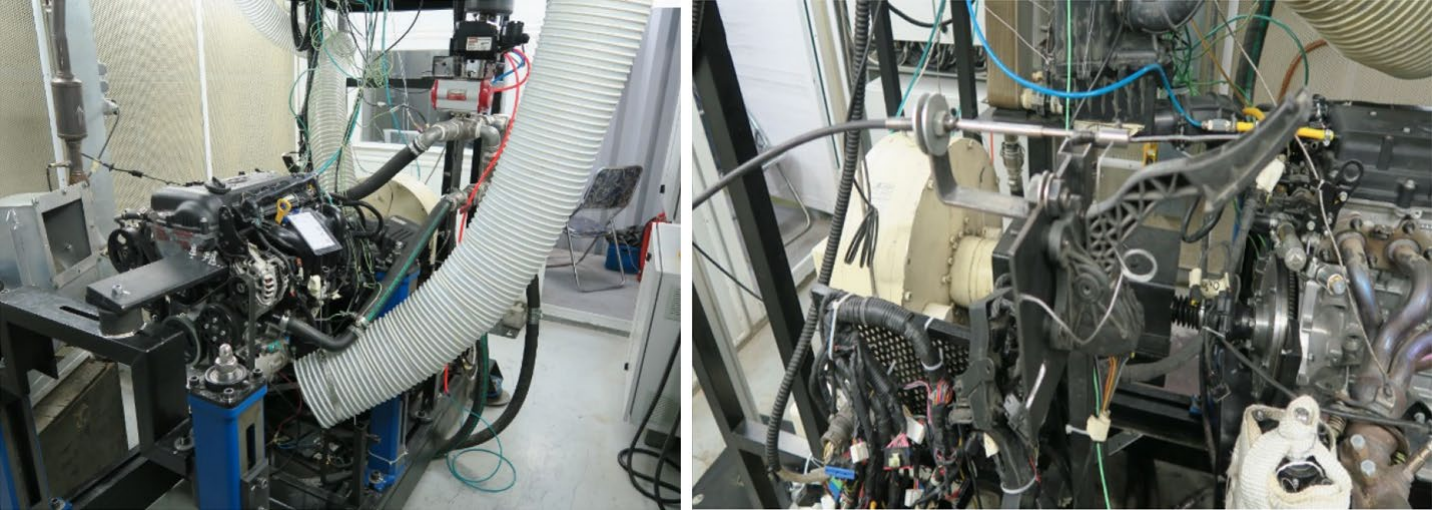
The KIA Cerato engine on test bed

This study used a KIA Cerato engine, as demonstrated in the engine test room in Fig. 1. The technical specification of KIA Cerato engine is provided in Table 1.

**Table 1:** Specifications of KIA Cerato engine

Parameter	Unit	Value
Bore	mm	86
Stroke	mm	86
Connecting rod length	mm	143.5
Number of cylinders		4
Displacement	cc	2000
Maximum power	kW	92
Maximum speed	RPM	7000
Rated speed	RPM	6000
Compression ratio		10.5

To obtain the experimental data, the engine coupled with an eddy current dynamometer was tested and the engine torque and BSFC data were collected. The collected data were used to validate the AVL model, as described in next section.

The Schenck 190 kW dynamometer was used in engine test room for running the engine and collecting data at various conditions. In addition, the uncertainty of each measuring instrument is provided in Table 2.

**Table 2:** Uncertainties of measuring instruments

Parameter	Unit	Measuring equipment	Nominal value range	Uncertainty	Relative uncertainty/ %
Air temperature	°C	Dina engine connect [46]	0–100	$\pm 2$	2
Air pressure	kPa	Dina engine connect [46]	0–100	$\pm 1$	1
Relative Humidity	%	Dina engine connect [46]	5–95	$\pm 2.5$	2.63
Fuel temperature	°C	Dina engine connect [46]	0–100	$\pm 0.2$	0.2
Engine speed	RPM	Schenck 190 kW dynamometer [46]	100–7000	$\pm 4$	0.06
Engine torque	Nm	Schenck 190 kW dynamometer [46]	0–250	$\pm 0.95$	0.38
Engine power	kW	Schenck 190 kW dynamometer [46]	0–190	$\pm 1.2$	0.63
Engine fuel consumption	kg h^−1^	Dina fuel mass flow meter [46]	1–50	$\pm 0.25$	0.5

### Engine mathematical model

**Fig. 2:**
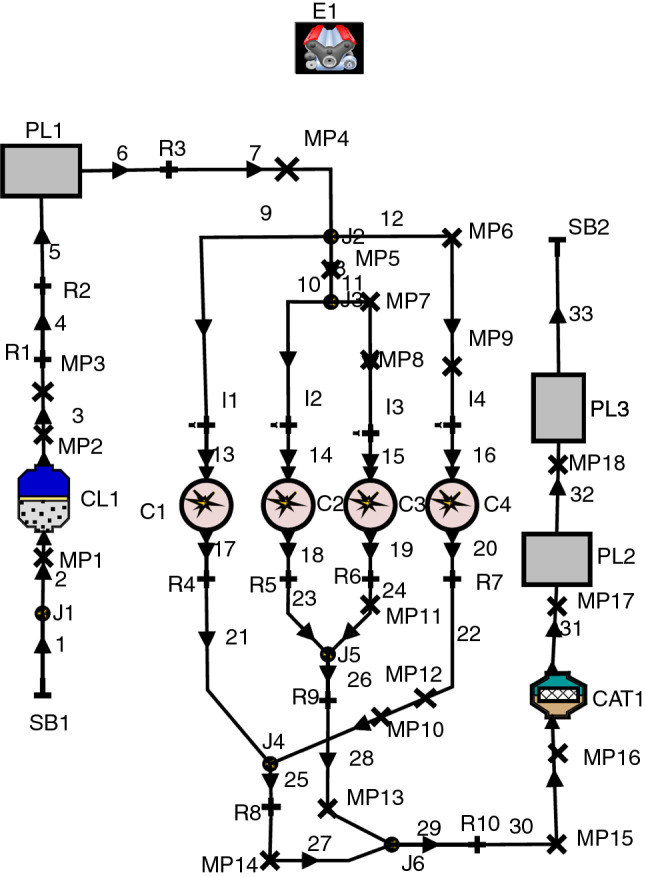
Block diagram of engine model in AVL BOOST software

The AVL BOOST is a 1D thermodynamic simulation software for numerical modeling of internal combustion engines. In this paper, this software was used to model the tested engine. The model of our naturally aspirated Kia engine is shown in Fig. 2. As can be seen in Fig. 2, this model consists of four cylinders (C1, C2, C3 and C4 blocks) with four fuel ports (I1, I2, I3 and I4 blocks). AC1 and CAT1 are the air cleaner and catalyst unit components in this figure.

#### The combustion model

The Vibe two zone combustion model was used for calculating the rate of heat release in mathematical model [47]. In this combustion model, the combustion chamber is divided into two zones: burnt and unburnt zones. Therefore, the temperatures of aforementioned zones are calculated separately and the fraction of unburned can be calculated precisely by using such combustion model. Fuel mass burned fraction (*x*) during combustion in Vibe two zone model is expressed as below:

1 $$x = 1 - { \exp }\left[ { - a\left( {\frac{{ \propto - {\text{SOC}}}}{\text{BDUR}}} \right)^{{\text{m}} + 1} } \right],$$

where SOC, BDUR, $\propto$, *m* and a are the start of the combustion, burn duration, crankshaft angle, Vibe shape and Vibe parameter, respectively. Vibe shape parameter indicates the position of the brunt for the combustion position. Also, Woschni model has been used for modeling of the heat transfer between gas and cylinder walls [48, 49].

### Air Brayton cycle

**Fig. 3:**
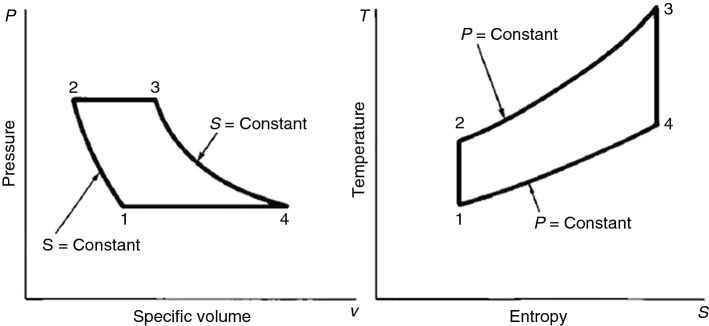
P–V and T–S diagrams of a standard air Brayton cycle

The air Brayton cycle is a famous thermodynamic cycle consisting of four thermodynamic processes: compression of air in compressor (1 to 2), heating air in a heat exchanger (2 to 3), expansion of heated air in the turbine (3 to 4) and cooling the air at constant pressure (4-1) [50]. The P–V and T–S diagrams of ABC are shown in Fig. 3. Brayton cycle is the standard cycle of the gas turbine, and it is used widely in many industries as power production heat engines such as powerplants, airplanes and multi-generation systems [50].

**Fig. 4:**
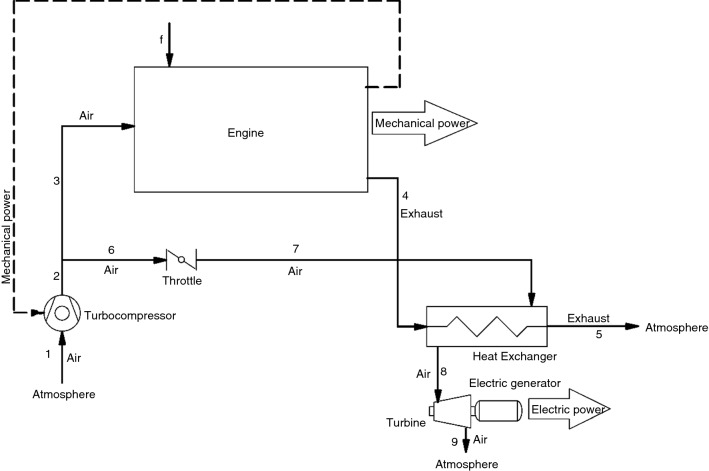
Block diagram of turbo-compound system

In this study, the air Brayton cycle (ABC), shown in Fig. 4, was coupled to KIA Cerato engine for WHR. A turbo-compressor was added to engine for two purposes: charging extra air to engine (supercharging) and charging air to ABC. The fraction of charged air entering ABC was controlled by a throttle [51–53] installed before ABC heat exchanger. In fact, this throttle controls the rate of air shared between engine and ABC. When it is closed, no air will enter the ABC; therefore, all of the charged air will be transferred to engine to provide the maximum power. When the throttle is fully open, a small fraction of charged air is transferred to ABC and it is warmed up by absorbing heat energy from the engine exhaust in the heat exchanger. Then, it will be expanded in turbine to generate electrical power.

**Fig. 5:**
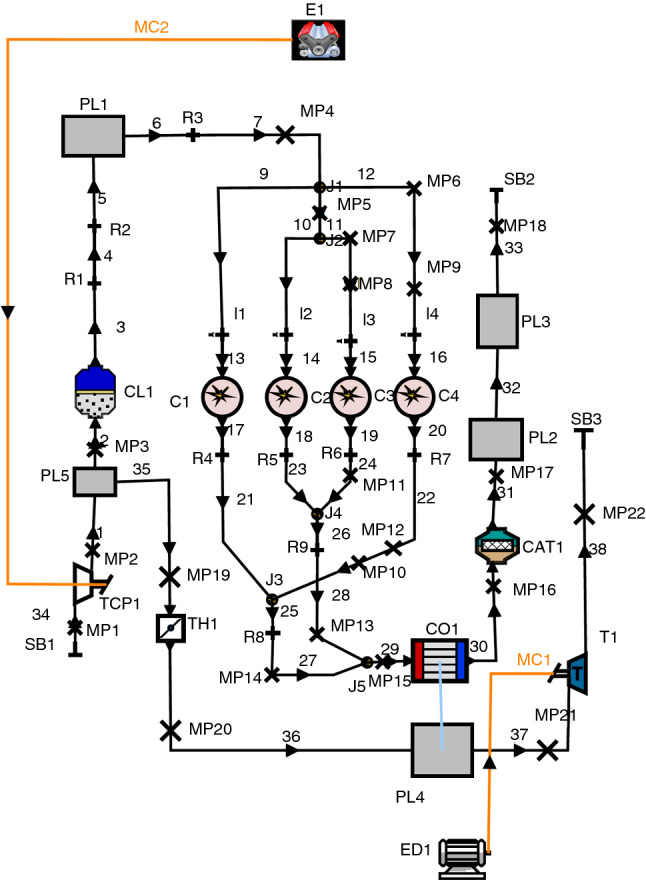
Block diagram of turbo-compound system in AVL BOOST software

Figure 4 presents a schematic diagram of the proposed WHR system. As shown, the ABC recovered energy is proposed to produce extra electric power, while the engine is boost up by the turbo-compounding. The proposed system model is also shown in AVL BOOST software in Fig. 5. TC1, T1 and TH1 are the turbo-compressor, turbine and throttle components. CO1 block is the heat exchanger where the engine exhaust flows and some of the wasted heat is transferred to cold flow via PL4 block. The specifications of the added turbo-compressor and turbine used in this model are shown in Table 3. In addition, ED1 component in AVL model is the electric generator which converts turbine mechanical power to electric power, and it is also shown in Fig. 4 as electric generator block.

**Table 3:** The specifications of turbo-compressor (TC) and turbine

Parameter	Value
TC pressure ratio	1.2
TC isentropic efficiency	0.7
TC mechanical efficiency	0.9
Turbine equivalent discharge coefficient	0.045
Turbine isentropic efficiency	0.8
Turbine mechanical efficiency	0.98

### Exergy analysis

The data required for exergy analysis were extracted from AVL software, and the engine thermodynamic model for exergy analysis was developed in MATLAB software. In exergy analysis, the air was assumed to be ideal gas in the model. In addition, the energy and exergy analysis of the system has been performed under the steady-state condition. The exergy balance concept was used to find the exergy destruction. The exergy destruction expressions for the main components of the system are presented in Table 4.

**Table 4:** The exergy destruction expression for each component [16, 27, 39]

Components	Expressions
Engine	$I_{\text{engine}} = \dot{m}_{3} e_{3} + \dot{m}_{f} e_{f} - \dot{m}_{4} e_{4} - \dot{E}_{\text{ht}} -\dot{W}_{\text{engine}}$
Turbo-compressor	$I_{\text{TC}} = \dot{m}_{1} e_{1} - \dot{m}_{2} e_{2} + \dot{W}_{\text{TC}}$
Heat exchanger	$I_{\text{HEX}} = \dot{m}_{4} e_{4} - \dot{m}_{5} e_{5} + \dot{m}_{7} e_{7} - \dot{m}_{8} e_{8}$
Turbine	$I_{\text{Turbine}} = \dot{m}_{8} e_{8} - \dot{m}_{9} e_{9} - \dot{W}_{\text{Turbine}}$

The exergy of fuel ($e_{\text{f}}$) and specific exergy of engine exhaust gas ($e_{4}$) can be calculated from equations below [16, 27, 39]:

2 $$e_{\text{f}} = \left( {1.0401 + 0.1728\frac{h}{c} + 0.0432\frac{o}{c} + 0.2169\frac{s}{c}\left( {1 - 2.0628\frac{h}{c}} \right)} \right){\text{LHV}},{\text{kJ}}\,{\text{ kg}}^{ - 1}$$

3 $$e_{4} = e_{{{\text{ph}}4}} + e_{{{\text{ch}}4}} ,\, {\text{kJ}}\,{\text{ kg}}^{ - 1} ,$$

where h, c, o, s, LHV, $e_{{{\text{ph}}4}}$ and $e_{{{\text{ch}}4}}$ are mass fractions of hydrogen, carbon, oxygen, sulfur contents, lower heating value of fuel, physical and chemical specific exergy of exhaust gas at stream 4, respectively. The expressions for calculation of physical and chemical exergies are:

4 $$e_{{{\text{ph}}4}} = \left( {h_{4} - h_{0} } \right) - T_{0} \left( {s_{4} - s_{0} } \right),{\text{kJ }}\,{\text{kg}}^{ - 1}$$

5 $$\bar{e}_{{{\text{ch}}4}} = \bar{R}T_{0} \sum a_{{\text{i}}} \ln \frac{{x_{{\text{i}}} }}{{x_{0} }},{\text{kJ}}\,{\text{ kmol}}^{ - 1},$$

where $x_{\text{i}}$, $x_{0}$, $\bar{R}$ and $T_{0}$ are the mole fraction of x component, mole fraction of x component in the environment, international gas constant and temperature of reference condition, consecutively. The exergy rate of engine heat transfer to cooling jacket ($\dot{E}_{ht}$) is calculated as follows:

6 $$\dot{E}_{\text{ht}} = \left( {1 - \frac{{T_{0} }}{{T_{\text{c}} }}} \right)\dot{Q}_{\text{c}} ,{\text{kW}},$$

where $T_{\text{c}}$ and $\dot{Q}_{\text{c}}$ are the average temperature of the engine coolant and the rate of heat transfer to it, respectively. The reference condition for exergy analysis and the mole fraction of the air components in the environment are provided in Tables 5 and 6. The equations for calculation of system energy and exergy efficiencies are provided in Eqs. 7 and 8, respectively.

**Table 5:** The reference conditions for exergy analysis [39]

Parameter	Unit	Value
Temperature (${\mathbf{T}}_{0}$)	K	300
Pressure (${\mathbf{P}}_{0}$)	bar	1.01325

**Table 6:** The mole fraction of each air component in the environment [39]

Components	Mole fraction/%
O_2_	20.35
H_2_O	3.03
N_2_	75.67
CO	0.007
CO_2_	0.0345
H_2_	0.00005
Other	0.90845

7 $$\eta_{\text{energy}} = \frac{{\dot{W}_{\text{engine}} + \dot{W}_{\text{Turbine}} - \dot{W}_{\text{TC}} }}{{\dot{m}_{\text{f}} {\text{LHV}}}}$$

8 $$\eta_{\text{exergy}} = \frac{{\dot{W}_{\text{engine}} + \dot{W}_{\text{Turbine}} - \dot{W}_{\text{TC}} }}{{\dot{m}_{\text{f}} e_{\text{f}} }}$$

$\dot{W}_{\text{engine}}$, $\dot{W}_{\text{Turbine}}$, $\dot{W}_{\text{TC}}$ and LHV are engine brake power, turbine output power, turbo-compressor and fuel lower heating value, consecutively.

## Validation

**Fig. 6:**
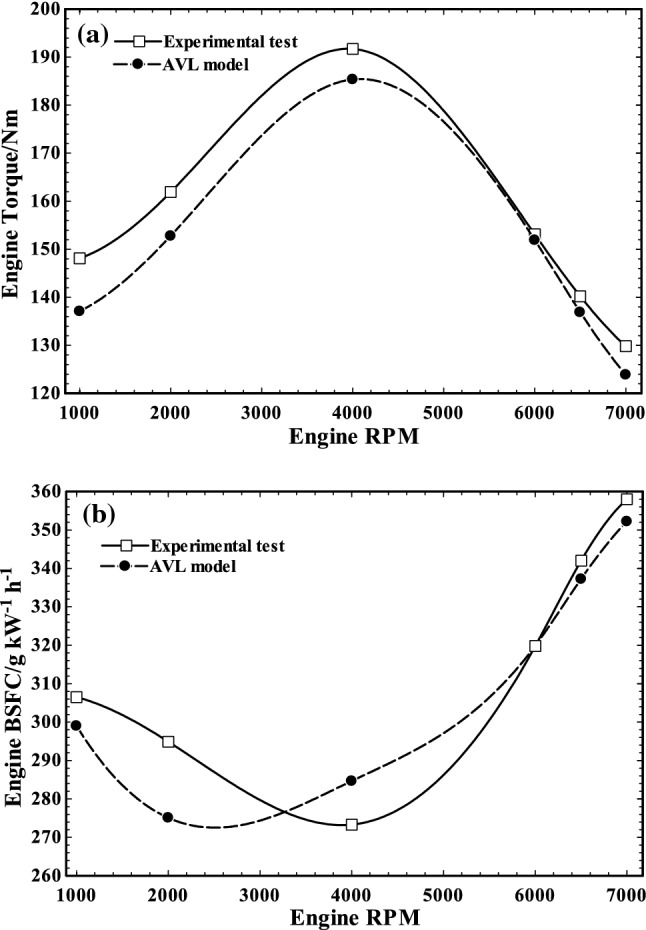
Comparison of engine torque (**a**) and BSFC (**b**) in various RPMs of AVL model with experimental tests

The experimental data obtained by testing the engine in the test bed were compared to AVL model data for validation of AVL model. Based on the results of comparison which are shown in Fig. 6, the maximum error of AVL model is below 8% (less than 5% in most of the speeds). Therefore, the model is valid and can represent the reality for further analysis.

## Result and discussion

The effects of adding ABC as a turbo-compounding system in engine at different parameters were evaluated by solving the AVL model in AVL BOOST software at rated condition (6000 RPM). The thermophysical properties at different measure points were then extracted from AVL and exported to MATLAB software for exergy analysis.

**Fig. 7:**
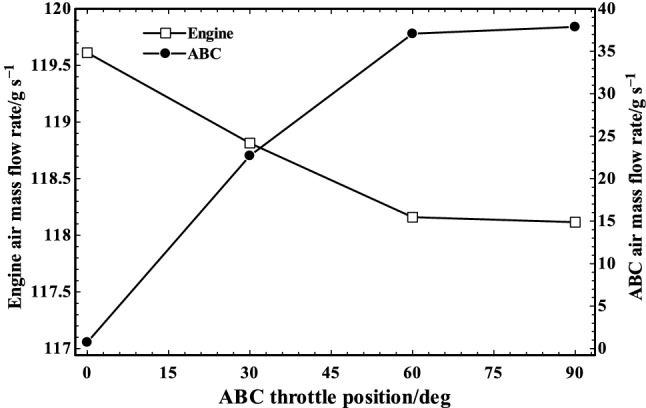
Engine and ABC air mass flow rates in various ABC throttle positions

Figure 7 shows the impacts of ABC throttle angles on engine and ABC air mass flow rates. The throttle was employed for controlling the mass flow rate of air between engine and ABC by providing a flexible system in which the amounts of electrical power generation of WHR system (ABC) and extra mechanical power produced by engine can be controlled and adjusted in different conditions. When throttle angle was at 0 degree (i.e., it was closed), no air passed through the ABC system and it was on standby. The rate of air mass flow through engine decreases by increase in the throttle angle which leads to charging some of the turbocharged air into the ABC cycle (see Fig. 7).

**Fig. 8:**
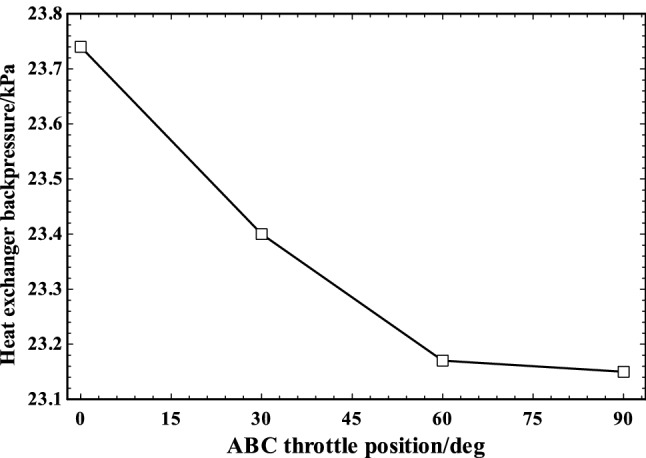
The heat exchanger backpressure various ABC throttle angles

The heat exchanger backpressure in various ABC throttle positions is shown in Fig. 8. The backpressure decreased from nearly 23.75 to 23.15 kPa by increment of ABC throttle angle from 0 up to 90°. The engine power loss due to backpressure burden on engine is calculated by AVL BOOST and applied in final calculations.

**Fig. 9:**
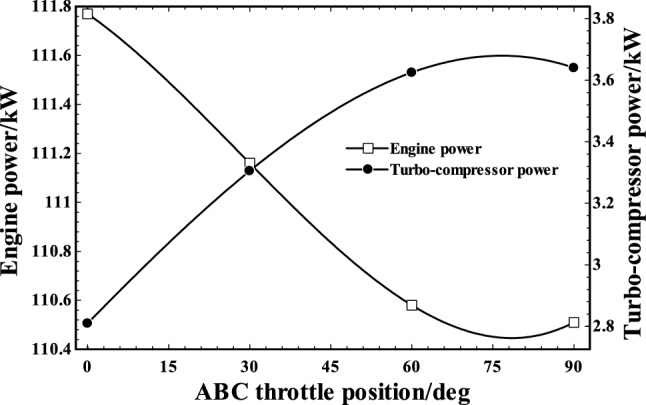
Engine power output and turbo-compressor power consumption in various ABC throttle positions

The variation of engine and turbo-compressor power at different ABC throttle positions is shown in Fig. 9. The engine mechanical power is changed from 111.8 kW to nearly 110.5 kW by changing the ABC throttle position between 0° and 90°. The engine power decreased minimally (1.16%) by increment of throttle angle which led to charging less air into the engine and more air into the ABC cycle. Furthermore, the turbo-compressor power consumption increased by increase in the throttle angle. This is due to the increment of backpressure in whole system, as more power is needed for turbo-compressor to compensate the backpressure of the installed ABC components.

**Fig. 10:**
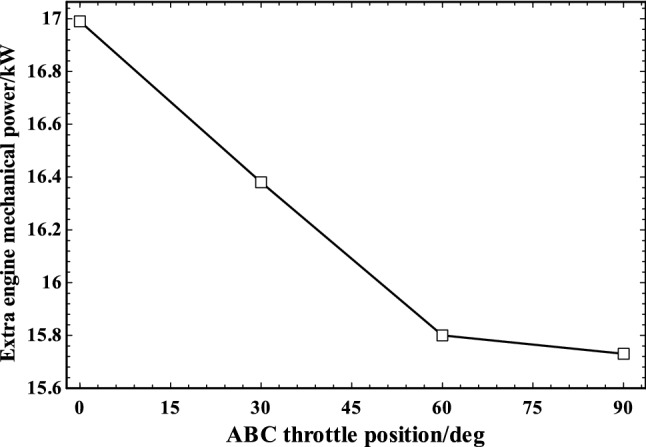
Extra engine mechanical power output in various ABC throttle positions

Figure 10 indicates the extra power available for the engine by installing the turbo-compressor which acts as a supercharging system at various ABC throttle angles. It should be noted that turbo-compressor power is provided mechanically from engine. The extra mechanical power of 17 kW was produced (~ 18% increase in engine power) by adding turbo-compressor compared to the original naturally aspirated engine when the ABC throttle was completely closed. AF ratio is assumed to be constant; therefore, by increase of the air mass flow rate, the fuel mass flow rate increases which results in production of more power by the supercharged engine. Furthermore, as shown in this figure, the generated extra power decreased from 17 to ~ 15.7 kW by changing the ABC throttle to fully open as the result of sharing of the compressed air between engine and ABC cycle.

**Fig. 11:**
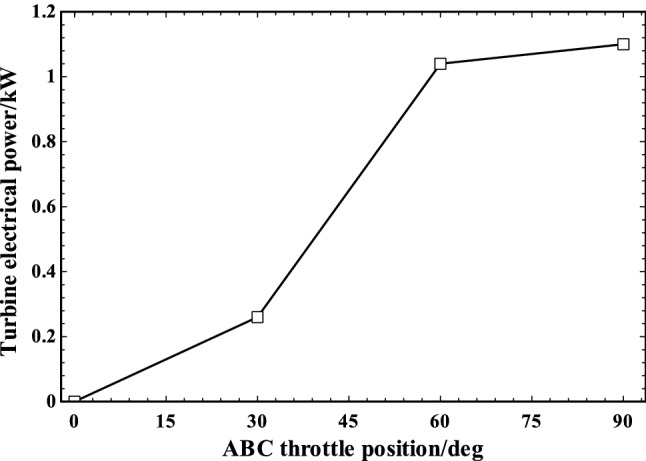
ABC turbine electrical power generation in various ABC throttle positions

The potential electrical power produced by ABC turbine at different ABC throttling positions is demonstrated in Fig. 11. There is a potential of recovering up to 1.1 kW from the exhaust at rated condition. The generated electrical power showed a rising trend by increase in the throttle angle due to the charging of more air into the ABC cycle for producing more electrical power.

**Fig. 12:**
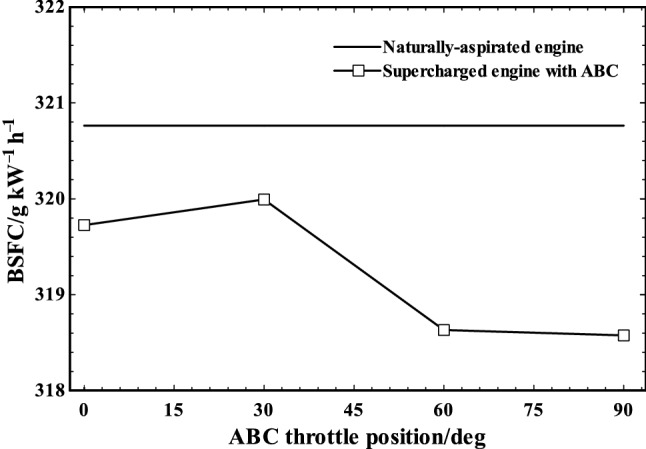
BSFC of naturally aspirated and supercharged engine at various ABC throttle positions

Figure 12 shows the BSFC variation of supercharged and naturally aspirated engines as compared to ABC throttle angle. As it can be seen, the BSFC increases slightly by increase in the throttle angle to 30° due to the reduction of engine power in supercharged engine. The minimal ABC power production will not overweight the engine power loss (due to the backpressure and lowering air mass flow rate) at this throttle position. On the other hand, the BSFC decreased to nearly 318.5 g kWh^−1^ when the throttle angle increased from 30 to 60°. The minimum of BSFC was happening at ABC throttle between 60 and 90° as demonstrated in Fig. 12. By comparing the naturally aspirated engine and supercharged engine (with ABC), it was found that engine BSFC was slightly improved (~ 1%) by adding the turbo-compound system.

**Fig. 13:**
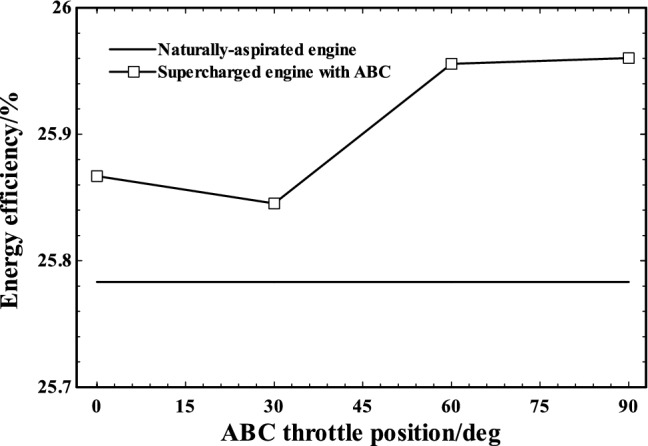
Energy efficiencies of naturally aspirated and supercharged engine at various ABC throttle positions

**Fig. 14:**
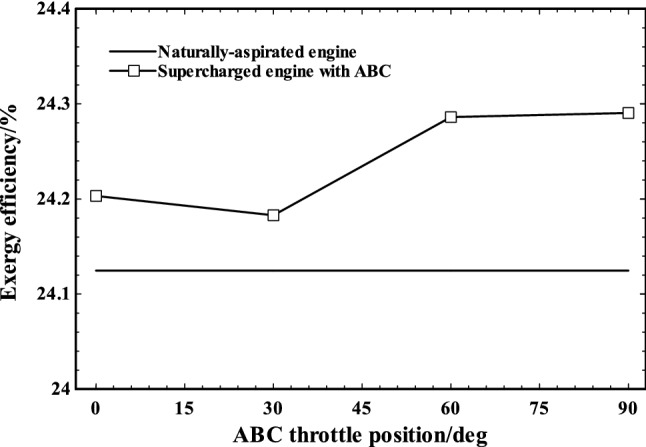
Exergy efficiencies of naturally aspirated and supercharged engine at various ABC throttle positions

Figures 13 and 14 indicate the exergy and energy efficiencies trends of naturally aspirated engine and supercharged engine (with ABC) by changing ABC throttling position. As shown, employment of turbo-compound system improved both the exergy and energy efficiencies slightly (nearly 1%) in supercharged engine compared to original naturally aspirated engine. Since employing supercharger and injecting more air corresponds to burning more fuel at constant air/fuel (AF) ratio, as expected, the exergy and energy efficiencies have not been changed considerably. Furthermore, by increase in ABC throttle angle, both exergy and energy efficiencies of system are increased. Therefore, activation of ABC system resulted in increase in supercharged engine energy and exergy efficiencies. It can be inferred from Fig. 14 that the amount of exergy improvement of turbo-compounding system is similar to previous study of turbo-compounding system conducted by Zi et al. [42].

**Fig. 15:**
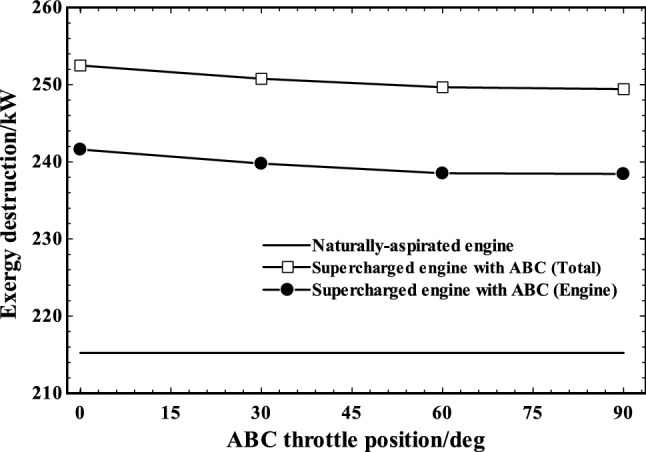
The Exergy destruction of whole system and engine in various ABC throttle angles

The exergy destruction is another important parameter demonstrating how efficient a component is operating and if there is a room for improvement. The exergy destruction of the total system including engine, turbo-compressor and ABC components as well as the engine block itself is presented in Fig. 15 and compared to original exergy destruction of the naturally aspirated engine. As shown, both engine and total exergy destruction were decreasing by ABC throttle position. In general, by adding more components, we should expect more total exergy destruction when comparing the original and modified engines. Comparing the engine block for naturally aspirated engine and supercharged engine, the exergy destructions of supercharged engine were moderately higher. It happened because extra air was transferred into the engine by supercharger resulted in injection of more fuel (at constant AF ratio) which led to increment of the exergy destruction.

**Fig. 16:**
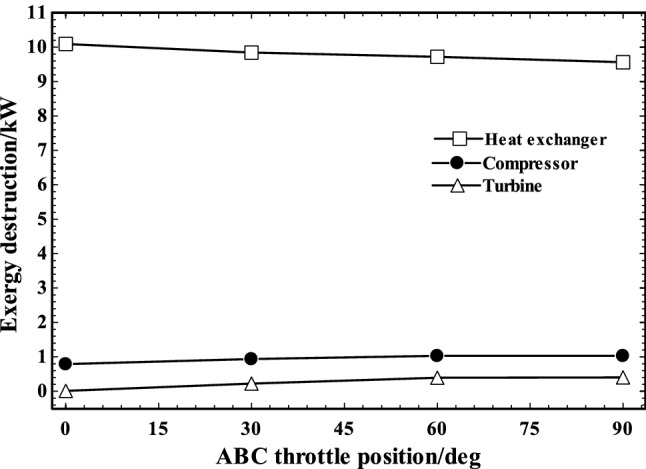
The exergy destruction of heat exchanger, compressor and turbine in various ABC throttle angles

Figure 16 demonstrates the exergy destruction for each of the ABC components at various throttle positions. The exergy destruction of the heat exchanger decreased slightly by the increase in ABC throttle angles due to the reduction in engine power production. However, a small increase in turbo-compressor and turbine exergy destructions was observed by increase in the ABC throttle angle which is resulted from increase in ABC charging air and power generation.

## Conclusions

In this paper, the energy and exergy analysis of installing a novel turbo-compound system for supercharging and mild hybridization of a naturally aspirated engine was performed using a KIA Cerato engine which was modeled numerically in AVL BOOST software. The results of modeling were compared to experimental data for validation. Additionally, the exergy model of whole system was developed to better understand the engine performance. The ABC was used in this study because it has less components and complexity compared to widely used ORC system; therefore, it imposes lower additional mass and pressure drop on vehicle. The results have shown a slight increase in BSFC, energy and exergy efficiencies of supercharged engine compared to naturally aspirated engine by employment of ABC as waste heat recovery system. Furthermore, employment of ABC led to production of up to 1.1 kW extra power which can be used for parasitic loads or powering a battery in mild hybridization. The main conclusion drawn from this paper can be listed as:

- Adding turbo-compressor increased the engine power output approximately between 16 and 18%.
- The turbo-compressor power consumption increased by increment of ABC throttle angle
- The engine power output can be controlled by ABC throttle position and it reduced by about 1.3 kW when ABC throttle gets fully open.
- The ABC turbine was capable of producing up to ~ 1.1 kW electrical power at the engine rated speed.
- Adding turbo-compound system to engine resulted in small reduction in BSFC of about 1%.
- The system energy and exergy efficiencies increased slightly by adding turbo-compound system. This can be optimized by adjusting the AF ratio in future research.
- The average exergy destruction of engine, heat exchanger, turbo-compressor and turbine were 239, 9.9, 0.95 and 0.2 kW, respectively.
- The backpressure caused by installing the heat exchanger on engine exhaust system was between 23.1 and 23.8 kPa, at different throttle positions.

## List of symbols

ABC: Air Brayton cycle
*a*: Vibe parameter
BDUR: Burn duration (deg)
$\dot{\varvec{E}}$: Exergy rate (kW)
*e*: Specific exergy (kJ kg^−1^)
*I*: Exergy destruction (kW)
*m*: Vibe shape
$\dot{\varvec{m}}$: Mass flow rate (kg s^−1^)
ORC: Organic Rankine cycle
$\dot{\varvec{Q}}$: Heat transfer rate (kW)
SOC: Start of combustion (deg)
TC: Turbo compressor
WHR: Waste heat recovery
$\dot{\varvec{W}}$: Mechanical power (kW)
$\propto$: Crank shaft angle (deg)

## Subscripts

C: Coolant
ch: Chemical
f: Fuel
HEX: Heat exchanger
ht: Heat transfer
ph: Physical
